# Role of Mifepristone in Induction of Labor in Full-Term Pregnancy

**DOI:** 10.7759/cureus.71632

**Published:** 2024-10-16

**Authors:** Payal Boipai, Tulika Sinha, Shiwani Kumari, Pooja Kumari, Apoorwa Sharma, Kiran Trivedi

**Affiliations:** 1 Obstetrics and Gynecology, Rajendra Institute of Medical Sciences, Ranchi, IND; 2 Obstetrics and Gynecology, Bharti Vidyapeeth Medical College, Pune, IND

**Keywords:** active phase of labor, bishop score, dinoprostone gel, induction to delivery interval, labor induction, mifepristone, primigravida, term pregnancy

## Abstract

Objective

This study sought to evaluate the safety and efficacy of mifepristone as a uterine sensitizer in shortening induction to delivery time in term pregnancy.

Study design

A prospective study was carried out on primigravida with a singleton term pregnancy, cephalic presentation, 37 to 41 weeks gestation, Bishop score ≤6, and consented to the study. A total of 116 participants were divided into two groups by random computer-generated sequence. On admission, the Bishop score was assessed. Group A (n=58) received 200 mg of mifepristone. Group B (n=58) received a placebo orally. In both groups, a post-intervention assessment was done after 24 hours, intracervical dinoprostone gel was administered with a maximum of three doses, six hours apart, if the Bishop score was ≤6. The primary outcome was to evaluate the effectiveness of oral mifepristone based on Bishop score improvement, the need for dinoprostone gel, and induction to delivery time. The secondary outcome was to evaluate the safety of oral mifepristone based on cesarean section rate and fetomaternal outcome.

Results

The Bishop score markedly improved in group A after 24 hours of intervention. A total of 31 women delivered vaginally after receiving only mifepristone. Mean induction to delivery time significantly improved in group A at 23.22±12.57 hours as compared to that in group B at 38.79±7.32 hours. Cesarean delivery rate was lower in group A (27.59%) compared to group B (44.83%). Birth outcomes were consistent in both groups with no neonatal mortality.

Conclusion

Oral mifepristone has proved as a promising agent as a uterine sensitizer in inducing labor as it has significantly decreased induction to delivery time.

## Introduction

Induction of labor aims at achieving vaginal delivery by stimulation of the uterus before the onset of spontaneous labor [1]. The introduction of new oxytocics and techniques of induction, both of which are more effective and predictable, has significantly changed our traditionally conservative approach to labor induction [2]. Medications that ripen the cervix are essential in modern obstetrics. The Bishop score is the most widely used method for assessing cervical ripening due to its simplicity and high predictive value [3]. Progesterone suppresses myometrial contractions and maintains cervical competence throughout pregnancy. This provides the basis for exploring progesterone receptor antagonists as agents for cervical ripening. Mifepristone, a synthetic steroid hormone analog, has antiprogesterone and anti-glucocorticoid properties [4]. Mifepristone is used as a pretreatment to prime the cervix adequately [5]. It enhances uterine sensitivity to prostaglandins, thereby promoting labor facilitation. Prostaglandin E2 (PGE2), commonly referred to as dinoprostone, is a naturally occurring compound that helps promote labor [6]. It is commonly administered vaginally in the form of a gel, suppository, or insert. Limited studies have been carried out on the efficacy and safety of oral mifepristone. A current study has been conducted for the evaluation of the safety and efficacy of oral mifepristone as a uterine sensitizer for labor induction in primigravida.

## Materials and methods

A prospective cohort study was carried out in the Emergency Obstetrics unit of a tertiary care hospital, Rajendra Institute of Medical Sciences (RIMS), Ranchi, from September 2023 to June 2024. This study was conducted after receiving approval from the Institutional Ethics Committee, RIMS, Ranchi (Memo No. 30, dated February 8, 2023).

Study population

The inclusion criteria were primigravida women with a singleton live pregnancy and a gestational age from 37 weeks to 41 weeks, cephalic presentation, Bishop score ≤6, and consented to participate in the study. The exclusion criteria were women with multiple pregnancies, any obstetric complications, medical comorbidities like diabetes mellitus, cardiac disease, impaired hepatic or renal function, hematological disorders, history of uterine surgery, cephalopelvic disproportion, hypersensitivity to the drugs being administered, and fetal conditions like oligohydramnios, abnormal doppler studies, fetal growth restriction, intrauterine fetal demise, malpresentation, and malposition.

Sample size

A total of 116 pregnant women were divided into two groups, 58 in each. The sample size calculation was done based on a study by Gomathy et al. [6].

Sample size calculation:

n= {Z(1-a/2)}^2^ (S1^2 ^+ S2^2^)/d^2^

(Where "n" is the sample size, "S1" is the standard deviation of the first population, "S2" is the standard deviation of the second population, and "d" is the absolute precision on either side of the difference in prevalence.)

​​​​​Values of Z (1-a/2) = 1.96, d = 1, S1 = 1.5, S2 = 3.5, and d = 1.

n = (1.96)^2^ (1.5^2^ + 3.5^2^)/1

n = 58

Sampling technique

**Figure 1:**
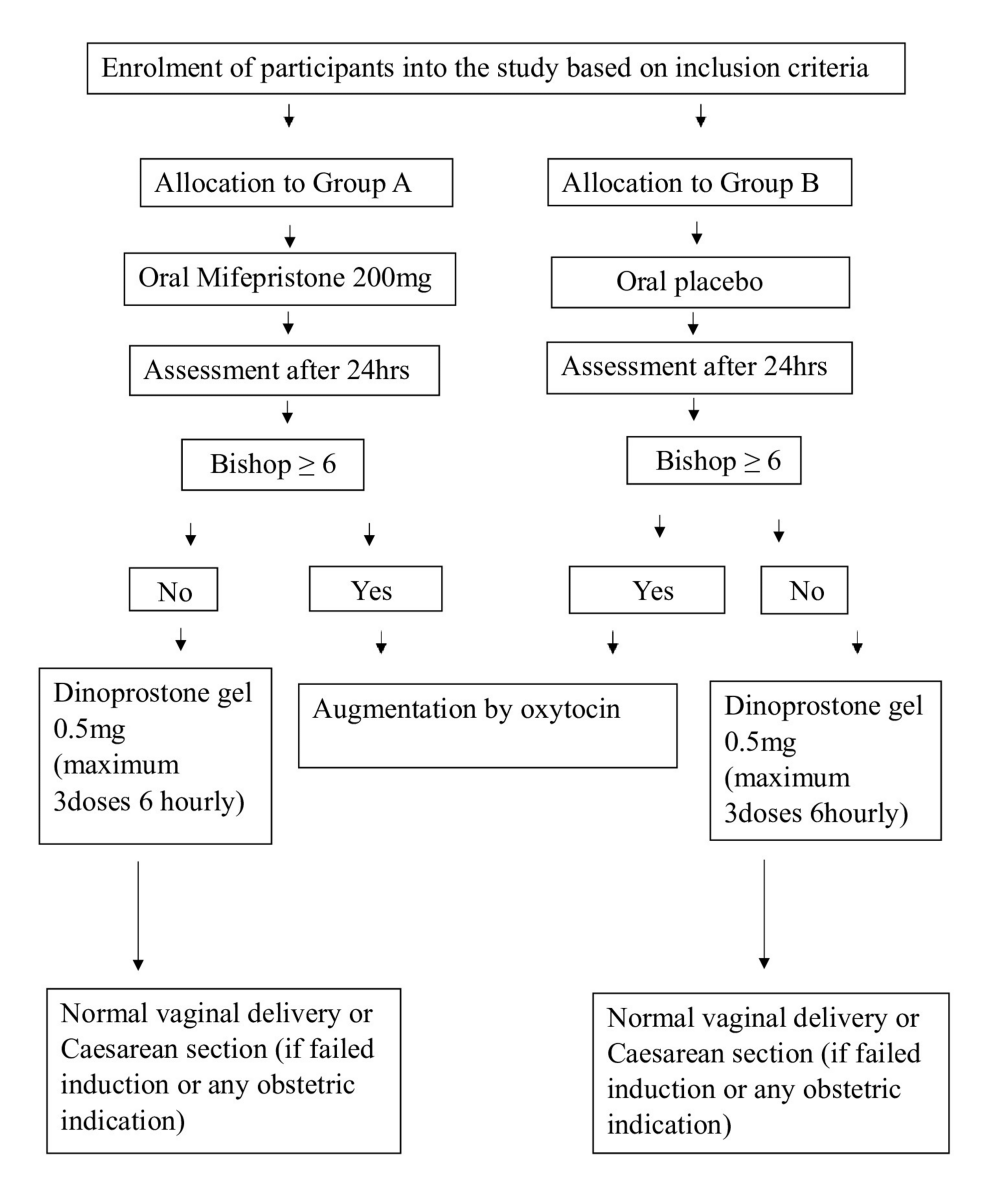
Study design

Random allocation of 116 participants was done according to the computer-generated sequence. They were divided into two treatment groups; group A received oral mifepristone 200 mg on admission, while group B received a placebo on an inpatient basis (Figure 1). The cervix was assessed by modified bishop's score with dilatation, consistency, position and length of the cervix, and station of presenting part. A score ≤6 was considered an unfavorable cervix and the participants were included if they had a score ≤6. The initial assessment was conducted after 24 hours, the progress of labor was assessed and dinoprostone gel was applied, if Bishop score ≤6, for a maximum of three doses, six hours apart. Progress of labor was monitored by a modified partograph and fetal well-being assessment was done by cardiotocography. The socio-demographic profile, gestational age, improvement in the bishop score, induction-to-delivery time interval, and feto-maternal outcomes were studied, recorded in a proforma, compiled, and analyzed.

Data analysis

A standard template was made in Microsoft Excel 16 (Microsoft Corp., Redmond, WA, USA) to collect data and it was analyzed in IBM SPSS Statistics for Windows, Version 25 (IBM Corp., Armonk, NY, USA). Qualitative data were expressed in percentages and proportions whereas quantitative data in mean, standard deviation, and confidence interval parametric or non-parametric tests were applied as per the nature of the data and p-value.

## Results

Demographic profile of groups A and B

The mean age of women was 22.93±3.70 years and 23.27±3.67 years in groups A and B, respectively (Table 1). The majority of them were booked in both the mifepristone (68.90%) and dinoprostone (74.13%) groups. Women were predominantly from urban backgrounds in both the mifepristone (84.48%) and dinoprostone (87.93%) groups. No statistical differences were observed between the two groups concerning age, residence, and booking status.

**Table 1:** Comparison of the demographic profile of the participants Booking status: According to the World Health Organization definition of "booking status," a pregnant woman is said to be "booked," if she has had at least four antenatal check-ups after being registered and confirmed to be pregnant [7].

Characteristic		Group A	Group B	P-value
Mean age±SD (years)		22.93±3.70	23.27±3.67	0.641
Residence	Urban	49 (84.48%)	51 (87.93%)	0.788
	Rural	9 (15.51%)	7 (12.06%)	
Booking status	Booked	40 (68.90%)	43 (74.13%)	0.681
	Unbooked	18 (31.03%)	15 (25.80%)	

Period of gestation on admission

**Figure 2:**
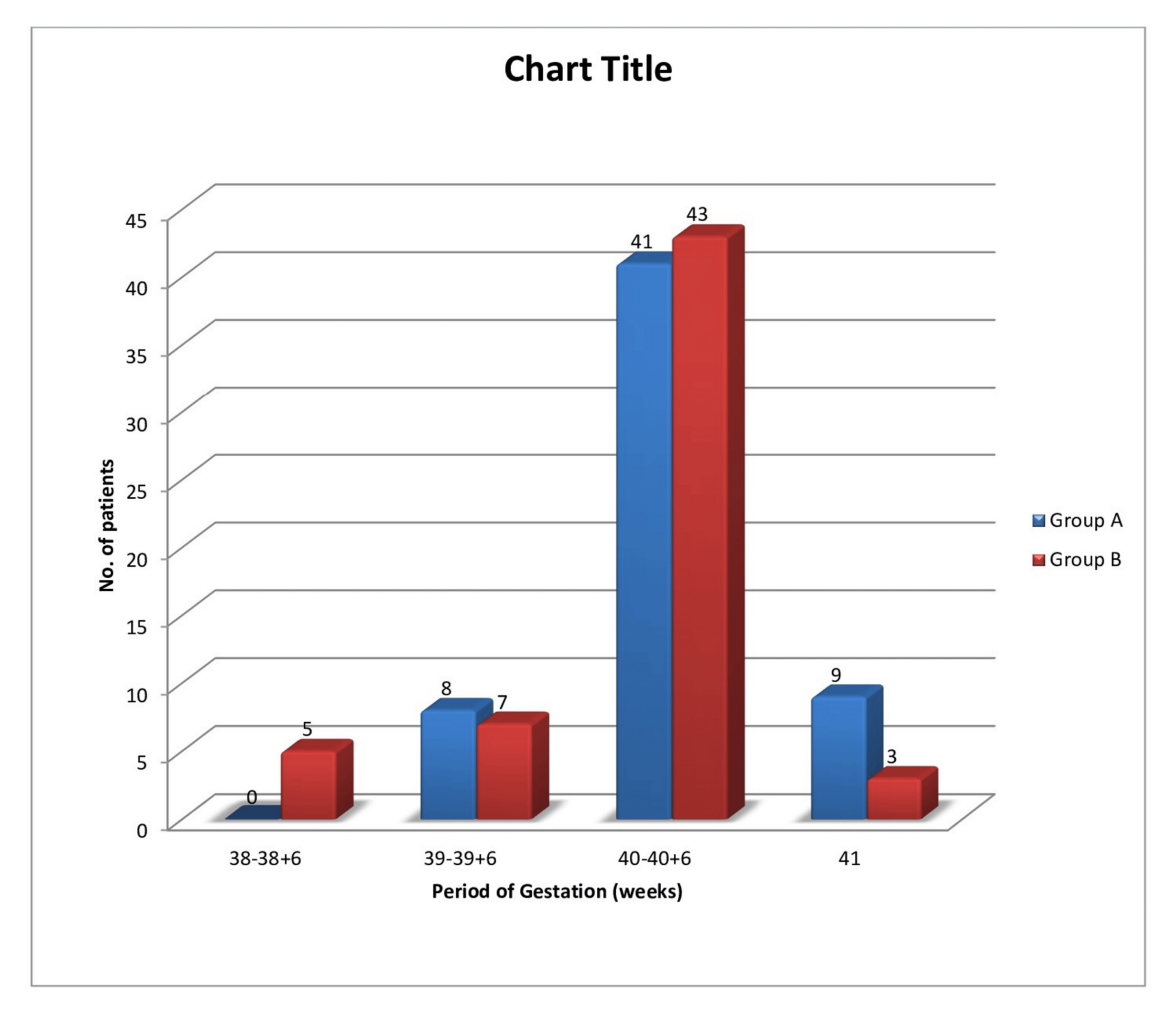
Comparison of period of gestation at the time of admission in both groups

In group A, 70.69% of patients had a gestational age between 40 weeks to 40 weeks+6 days, followed by 15.52% of patients in 41 weeks of gestation and 13.79% of patients in 39 weeks to 39 weeks+6 days (Figure 2). In group B, 74.14% of patients had a gestational age between 40 weeks to 40 weeks+6 days, followed by 12.07% in 39 to 39+6 weeks of gestation, 8.62% in 38 to 38+6days, and 5.17% in 41 weeks of gestation.

Changes in Bishop score

The mean Bishop score, 24 hours after the first intervention was 4.81 ± 0.73 and 4.43 ± 0.53 in groups A and B respectively, with statistically significant improvement of Bishop score (p-value 0.021) (Table 2).

**Table 2:** Improvement of Bishop score

Bishop score	Group A Mean± SD (hours)	Group B Mean± SD (hours)	P-value
At admission	4.34±0.68	4.37±0.52	0.962
After 24hours of first intervention	4.81±0.73	4.43±0.53	0.021 (S)

Number of gel used for labor induction

In this study, 31 women (53.45%) in the mifepristone-treated group went into labor and had a vaginal delivery without requiring dinoprostone gel (Table 3). The above observations show a statistically significant p-value to be <0.0001.

**Table 3:** Number of dinoprostone gel used for induction of labor

No. of dinoprostone gel used	Group A		Group B	
	N	%	N	%
0 (No gel applied)	31	53.45	0	0.00
1	9	15.52	8	13.79
2	11	18.97	30	51.72
3	7	12.07	20	34.48
Total	58	100.00	58	100.00

Induction to delivery interval

The mean active phase to the delivery interval was 4.34±1.44 hours and 3.59±1.64 hours in groups A and B respectively, this interval has decreased in group B, which is statistically significant (p-value 0.018) (Table 4). The mean induction-to-delivery interval was 23.22 ± 12.57 hours in group A and 38.79 ± 7.32 hours in group B. Overall induction to delivery interval has shown promising results in the mifepristone-treated group with a p-value < 0.0001.

**Table 4:** Induction to the delivery time interval in both groups

Parameters	Group A Mean±SD (hours)	Group B Mean±SD (hours)	P-value
Duration between induction to active phase of labor	7.58±6.36	8.97±4.31	0.071
Duration between active phase to delivery interval	4.34±1.44	3.59±1.64	0.018 (S)
Induction to delivery time interval	23.22±12.57	38.79±7.32	<0.0001 (S)

Comparison of maternal outcomes

The rate of cesarean deliveries was comparatively less in group A (27.59%) (16 patients) than in group B (44.83%) (26 patients) (Table 5). In group A, 18.75% (3) patients developed fever, 12.50% (2) had a postpartum hemorrhage and 6.25% (1) had an unhealthy wound. In group B, 7.69% (2) patients developed fever, 7.69% (2) patients had unhealthy wounds and 3.85% (1) had postpartum hemorrhage. The average duration of hospital stay was 3.03 ± 1.54 days and 3.62 ± 1.75 in group A and group B respectively.

**Table 5:** Maternal outcomes NVD: normal vaginal delivery, LSCS: lower segment cesarean section

Parameters		Group A		Group B		P-value
		Number	%	Number	%	
Mode of delivery	Assisted vaginal delivery	2	3.45	0	0.00	0.071
	NVD	40	68.97	32	55.17	
	LSCS	16	27.59	26	44.83	
Complications	Fever	3	18.75	2	7.69	0.676
	Post partum hemorrhage	2	12.50	1	3.85	
	Wound inspection	1	6.25	2	7.69	
	Mean duration of hospital stay (days)	3.03±1.54		3.62±1.75		

Comparison of neonatal outcomes

In both groups, 8.62% of neonates were admitted to the neonatal intensive care unit (NICU) in our study (Table 6). No neonatal mortality in either group was found.

**Table 6:** Neonatal outcomes NICU: neonatal intensive care unit

Parameters	Group A		Group B		P-value
	Number	%	Number	%	
NICU admission	5	8.62	5	8.62	1.000
Mortality	0	0.00	0	0.00	

## Discussion

The average age of women was 22.93±3.70 years and 23.27±3.67 years in groups A and group B respectively; whereas the mean age was 24.91±3.35 years in a study by Jindal et al. (2019) [8]. The majority of them were booked in both groups, with 68.90% and 74.13% in groups A and B respectively. Women were predominantly from urban backgrounds in both groups, with 84.48% and 87.93% in groups A and B respectively, contrary to a study by Gupta et al. (2018) where the majority of patients belonged to rural areas in both groups [2]. Observations showed no statistical correlation between the two groups concerning age, residence, and booking status.

In group A, 70.69% of patients had a gestational age between 40 weeks to 40 weeks+6 days, followed by 15.52% of patients in 41 weeks of gestation and 13.79% of patients in 39 weeks to 39 weeks+6 days. In group B, 74.14% of patients had a gestational age between 40 weeks to 40 weeks+6 days, followed by 12.07% in 39 to 39+6 weeks of gestation, 8.62% in 38 to 38+6days, and 5.17% in 41 weeks of gestation. This result aligned with a study by Gomathy et al. (2022) [6].

The mean Bishop score post-intervention was 4.81±0.73 in group A and 4.43±0.53 in group B, indicating a significant (p-value 0.021) in the mifepristone group. In a study by Gomathy et al. (2022), a significant improvement in the Bishop score was observed in the group treated with mifepristone, with a p-value <0.004 which favors the findings of our study [6]. Compared to our study; Jindal et al. (2019) reported a dramatic improvement with both interventions, but the enhancement was statistically greater in women who received induction with dinoprostone gel (6±1.83) [8]. A similar observation was noted in a study done by Sailatha et al. (2017) showing a better Bishop score in the dinoprostone-treated group {4.7(±1.49)} as compared to the mifepristone-treated group {4.0(±1.48)} [9].

Successful induction of labor was found in 53.45% of patients in group A who went into labor and had a vaginal delivery without requiring dinoprostone gel, showing a statistically significant p-value as <0.0001; thus, indicating that mifepristone is a promising uterine sensitizer for induction of labor and to expedite the onset of labor and delivery. In a study by Sailatha et al. (2017), in the mifepristone group, 40% of women went into labor and had a vaginal delivery without the need for dinoprostone, with 30% of these women delivered within 24 hours [9].

The mean active phase to the delivery interval was 4.34±1.44 hours and 3.59±1.64 hours in groups A and B respectively. This indicates that patients treated with dinoprostone gel experienced a shorter duration from the active phase to delivery, which is statistically significant in favour of the dinoprostone gel administration group (p-value 0.018). In a study by Gomathy et al. (2022), there was no difference in the interval from the active phase to delivery [6]. In our study, the mean time taken from induction to delivery was 23.22 ± 12.57 hours in group A, compared to 38.79 ± 7.32 hours in group B. Overall, the time interval from induction to delivery was shorter in the mifepristone group compared to the dinoprostone-treated group, and this difference was statistically significant (p < 0.0001). On the contrary, Sailatha et al (2017) reported that the mean induction-to-delivery time interval was longer in the group with mifepristone intake (20.3 ± 1.5 hours) compared to the dinoprostone-treated group (11.5 ± 8.7 hours), with this difference being statistically significant with a p-value of 0.001 [9].

The rate of cesarean deliveries was comparatively less in group A (27.59%) than in group B (44.83%), though no statistical significance was found. This contrasts with the study by Gomathy et al. (2022), which found that 38.4% of patients in Group I required a cesarean section, compared to only 10.2% in Group II [6]. In a study by Sailatha et al. (2017), the number of women who had vaginal deliveries and cesarean sections was similar in both groups [9].

In our study, no serious side effects were detected in either group. In group A, minor side effects such as fever, cervical tear/vaginal lacerations, and wound sepsis were 3, 2, and 1 respectively. In group B, the same type of minor side effects was observed in 2, 1, and 2 patients respectively. No statistically significant difference was observed in the groups.

In a study by Lata et al. (2018), 6% of patients had complaints of nausea and vomiting in the mifepristone group as compared to 2% in the control group; whereas, in our study, no one had complaints of nausea and vomiting [10]. Yelikar et al. (2014) reported hyperstimulation in 4% (two patients) in each group, tachysystole in 2% (one patient) in the study group, and 4% (two patients) in the control group [11]. No such occurrences were seen in our group.

The neonatal outcomes were similar in both groups, and there were no cases of neonatal mortality in either group. In a study by Sailatha et al. (2017), no perinatal death was observed [9]. In both groups, 8.62% of neonates were admitted to NICU in our study.

There are several limitations to this study. Firstly, we did not conduct the study in a larger group of subjects, which could contribute to a better assessment of results of the safety and efficacy of mifepristone. Secondly, this study was single-centered. Incorporating multi-centric data could have made the analysis more representative. Additionally, a double-blinded, randomized controlled trial having a larger sample size, including complications during pregnancy and labor should be conducted. A systematic review and meta-analysis should also be performed to evaluate maternal and fetal outcomes of mifepristone.

## Conclusions

Mifepristone is an effective and safe pharmacological agent for induction of labor and to decrease the rate of cesarean section, as it improves Bishop score, along with a reduction in the induction to a delivery time interval and need for additional oxytocic without any major maternal or neonatal adverse effects. So, mifepristone can be a safe alternative for labor induction, either with or without dinoprostone gel. 

So, overall from the present study, we conclude that mifepristone can be used as a safe and effective alternative for labor induction, with favorable maternal and fetal outcomes thus decreasing the rate of cesarean section in primigravida with term pregnancy.
